# Sexual and Reproductive Healthcare Needs of Refugee Women Exposed to Gender-Based Violence: The Case for Trauma-Informed Care in Resettlement Contexts

**DOI:** 10.3390/ijerph21081046

**Published:** 2024-08-08

**Authors:** Cherra M. Mathis, Jordan J. Steiner, Andrea Kappas Mazzio, Meredith Bagwell-Gray, Karin Wachter, Crista Johnson-Agbakwu, Jill Messing, Jeanne Nizigiyimana

**Affiliations:** 1 College of Social Work, University of Kentucky, Lexington, KY 40506, USA; 2 Independent Researcher, Princeton, NJ 08542, USA; jjsteiner@gmail.com; 3 Independent Researcher, Mesa, AZ 85201, USA; akappasmazzio@gmail.com; 4 School of Social Welfare, University of Kansas, Lawrence, KS 66045, USA; 5 School of Social Work, Arizona State University, Phoenix, AZ 85004, USA; karin.wachter@asu.edu (K.W.);; 6 T.H. Chan Medical School, University of Massachusetts, Worcester, MA 01655, USA; 7 Valleywise Center for Refugee & Global Health, Phoenix, AZ 85008, USA

**Keywords:** refugee, women, reproductive health, sexual violence, trauma-informed care, service-providers

## Abstract

This paper assesses literature regarding the sexual and reproductive healthcare (SRH) needs of resettled refugee women who experienced gender-based violence (GBV) and trauma-informed care (TIC) principles utilized among SRH service providers. A systematic search identified relevant studies published between 2000 and 2021; no articles found reflected both SRH and TIC principles among refugee women. The search was therefore separated into two aims: to review the literature about SRH needs for refugee women in resettlement countries who experienced GBV (Aim 1) and to examine the use of TIC principles in SRH care among women who experienced GBV (Aim 2). Thematic analysis of the articles identified key themes. Twenty-six articles were included in the analysis across both aims (Aim 1 = 8, Aim 2 = 18). Aim 1 articles shared three factors shaping the SRH needs of resettled refugee women: the centrality of violence and trauma; structural barriers to SRH care; and actions, practices, and resources for service providers. Aim 2 articles illustrated seven key principles of TIC used in SRH service provision, such as empowerment; trauma-specific services and integrated care; connection; safety; collaboration; identity culture and context; and trustworthiness. Resettled refugee women’s experiences of violence necessitate trauma-informed SRH health care. While there is limited peer-reviewed literature regarding TIC-SRH care for refugee women, the findings regarding the SRH needs of refugee women and the findings regarding the implementation of TIC in SRH collectively frame recommendations for how SRH can be infused with TIC. An example from practice, in the form of the Refugee Women’s Health Clinic, is included as an exemplar of TIC SRH principles in action for the health of resettled refugee women who have survived gendered violence.

## 1. Introduction

Gender-based violence shapes women’s experiences of forced migration [1], amplifying the critical need to address the sexual and reproductive health (SRH) care of refugee women in resettlement contexts. Refugees are “people who have fled war, violence, conflict, or persecution and have crossed an international border to find safety in another country” and resettlement “involves the selection and transfer of refugees from a state in which they have sought protection to a third state that has agreed to admit them—as refugees—with permanent residence status,” [2] (n.p.). Canada, the United States, and Sweden are the largest recipients of resettled refugees [3]. Refugee status and identities are fluid and complex, and people may or may not identify as “refugees” post-resettlement [4].

Women are exposed to gender-based violence throughout their forced migration trajectories [1,5]. During their journeys, women describe experiencing sexual violence perpetrated by smugglers or traffickers, police officers and coast guards, and men traveling in their group [5,6,7]. Women can experience intimate partner violence (IPV)—that is, physical, psychological, or sexual violence by their partners or ex-partners—before, during, and after-migration [8]. Throughout the forced migration journey, women encounter barriers to seeking safety, with accounts of receiving little or no help from formal systems [5,9,10,11].

### 1.1. Sexual and Reproductive Health

Refugee women describe a high incidence of severe and persistent physical and mental health consequences from their experiences of IPV, including symptoms associated with depression, anxiety, complex trauma, and post-traumatic stress disorder [12,13]. Women also experience negative SRH outcomes associated with experiences of sexual violence and IPV. Physical health outcomes include HIV and other sexually transmitted infections (STIs), pain during intercourse, the need for reconstructive surgery, and unplanned pregnancies [14,15]. Behavioral health outcomes included reluctance and distrust around safe sex practices and limited knowledge and use of contraceptive options [15,16,17]. Violence-related trauma negatively impacts fetal development and maternal health [18,19]. Pregnancy-specific health concerns for refugee women include maternal and postpartum mental health [20,21,22], adolescent pregnancy, and serious maternal health complications [23]. Sexual violence can also impact long-term neurobiologic responses to trauma [24].

In seeking cervical screenings, obstetric care, sexual health services, or other gynecological support, women refugees can encounter mistreatment, discrimination, and rights violations such as involuntary sterilization [23,25], stigma associated with sexual-violence related pregnancies [26,27], and familial resistance to care [14,15,28]. They may also face the challenge of geographical distance to services and support networks [29]. Normative practices in the country of resettlement that are not attuned to refugee women’s needs can also further traumatize, retraumatize, and marginalize refugee women [29].

### 1.2. Trauma-Informed Care

Given the co-occurrence of violence, trauma, and SRH care needs among refugee women, trauma-informed health care is paramount. Grounded in trauma theory [30], systems, services, and programs that are trauma-informed recognize the wide-spread nature and implications of trauma, including signs, symptoms, and conditions that inhibit or foster healing, while integrating that knowledge within every facet of service delivery [31,32,33,34]. Generally, trauma-informed care draws from seven fundamental principles: safety; trustworthiness; empowerment; connection; collaboration; identity, culture, and context; and trauma-specific services and integrated care [31,32,34,35]. Trauma-informed care focuses on organizational functioning and institutional processes as well as the services offered. Trauma-specific services that aim to treat trauma-related symptomology and/or behaviors (e.g., post-traumatic stress disorder, depression), often through evidence-based treatments [30], may be integrated within (or work in partnership with) trauma-informed systems [30].

### 1.3. Scoping Review Aims

The aims of this scoping review are two-fold. The first aim is to examine contextual factors that shape SRH needs and access to SRH care among refugee women in resettlement who have experienced IPV and/or sexual violence. The second aim is to identify the ways in which trauma-informed care (TIC) principles have informed SHR care for survivors of sexual violence and IPV.

## 2. Materials and Methods

To identify relevant studies, we followed established guidelines [36] and identified six databases: Academic Search Premier, CINAHL, Medline, PsychInfo, PubMed, and Web of Science (This review was registered as INPLASY202470003). We developed search term concepts and expanded each one with relevant corresponding terms as indicated in Table 1, which formed the basis of our search string. Empirical articles were included if they described original research (quantitative, qualitative, or mixed methods) and were published in English-language peer-reviewed journals between 2000 and 2021. Included articles for Aim 1 focused on sexual violence and/or IPV and SRH of refugee women and adolescent girls aged 14 years or older in the context of SRH services in resettlement countries (exclusive of humanitarian, transition, or conflict settings); For Aim 2, articles included trauma-informed care principles named or discussed as part of a SRH intervention, program, or service model.

**Figure 1:**
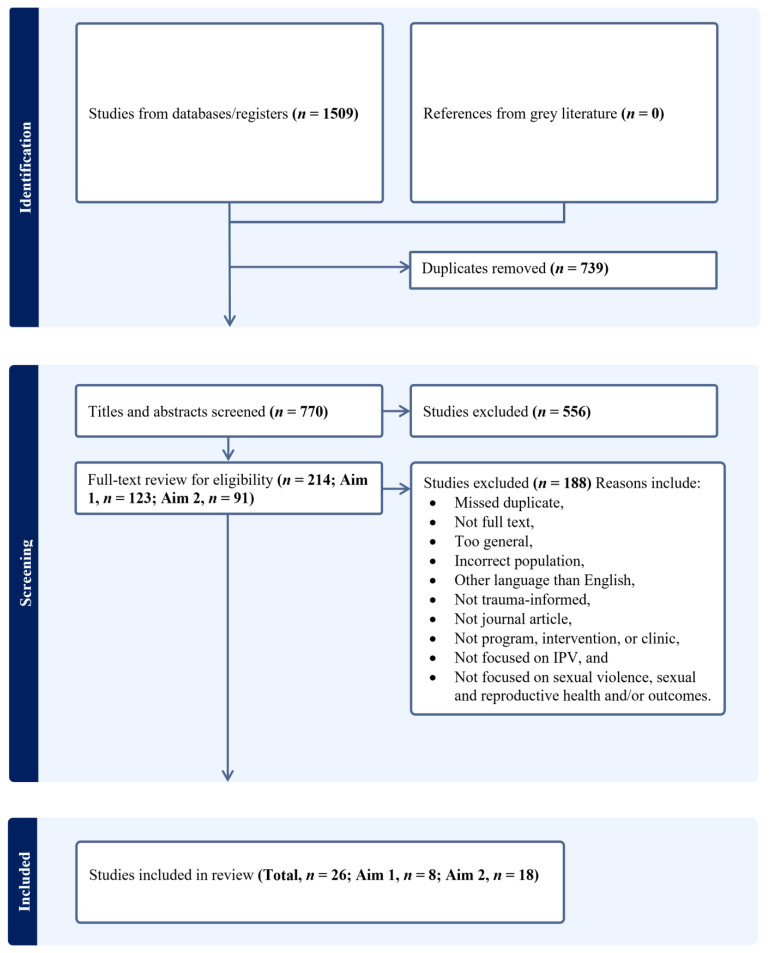
Sexual and Reproductive Healthcare Needs of Refugee Women Search Results.

The search yielded a total of 1509 potentially relevant articles. Of those articles, 739 duplicates were omitted. Another 556 were omitted by five of the study co-authors who screened titles and abstracts, with each title and abstract being screened by at least two team members. Five co-authors conducted a full-text review of the remaining 214 records (Aim 1, *n* = 123; Aim 2, *n* = 91), with two co-authors making decisions on each article. Articles with split decisions were resolved by a sixth team member or by group discussion. The final set of articles included *n* = 8 articles on SRH care needs for refugee women and *n* = 18 articles on trauma-informed SRH care for survivors of sexual violence and IPV (see Figure 1). We conducted a primary search in 2020 and a second search in 2022. We charted data from the 26 articles included in the final analysis to identify key themes from the findings of the articles. Related to our first study aim, we identified key themes in the articles on SRH needs for refugee women in resettlement contexts related to IPV or sexual violence. Repeated readings of the articles by two team members using a thematic analysis approach generated codes, which were refined and resolved into themes. For our second aim, themes were generated deductively based on established TIC principles [32,33,34,35] as well as barriers and facilitators to implementing trauma-informed care in sexual and reproductive health services.

## 3. Results

The findings from the analysis are organized according to the two study aims: (1) factors shaping SRH needs and access to SRH care among refugee women in resettlement who have experienced IPV and/or sexual violence, and (2) principles of trauma-informed care operationalized in sexual and reproductive health care for refugee women in resettlement. Articles for the first aim spanned countries of refugee resettlement, including the United Kingdom (*n* = 3), Canada (*n* = 2), the United States (*n* = 1), Switzerland (*n* = 1), and Sweden (*n* = 1). Articles for the second aim included the United Kingdom (*n* = 1), the United States (*n* = 14), Australia (*n* = 1), and South Africa (*n* = 2). An overview of the findings and the articles separated by aim, population, and theme can be found in Table 2 and Table 3.

### 3.1. Aim 1: Factors Shaping SRH Needs and Access to SRH Care among Refugee Women in Resettlement Who Have Experienced IPV and/or Sexual Violence

#### 3.1.1. The Centrality of Violence and Trauma

Power and Control in Reproduction and Bodily Autonomy. Three articles discussed power and control in reproduction and the lack of bodily autonomy among refugee women [37,38,39]. In the context of power and control, women’s partners controlled their reproductive choices, including whether to seek abortion care [37] or refuse to use contraception, such as condoms or birth control [38]. One of the reasons refugee women’s partners refused hormonal contraception was because they were afraid of the women gaining weight [38], which indicates an adherence to ascribed standards of feminine beauty. Partner-perpetrated reproductive coercion was associated with two different types of poor pregnancy outcomes: unwanted pregnancy and pregnancy loss from partner-perpetrated violence and abuse [38]. Refugee women’s experiences of reproductive coercion were also connected to their lack of control over the frequency of sexual intercourse [39]. Sexual intercourse was commonly seen as an obligation to male partners [39].

History of Sexual Violence. The history of IPV and sexual violence was commonplace among refugee patients [37,38,39,40,41,42,43] and illustrated how closely related experiences of sexual violence are to pregnancy as an outcome [40], as well as limiting contraceptive options as a tool of coercive control [39]. Thus, reproductive coercion and sexual violence were fundamentally linked. A common issue among women respondents in studies was their experience of vulnerability and shame that came with experiences of sexual violence and reproductive health outcomes, and the difficulties in speaking about their experiences of sexual violence [40,41]. Additionally, there was a cultural shame linked to sexual violence and a fear of parental reactions [37]. Moreover, migrants, refugees, and asylum seekers articulated that service providers did not fully understand the complexity of their distress or the difficulties and challenges exacerbated by sexual violence through their migration journey [37,40,42]. As a result of sexual violation and abuse, refugee participants identified two primary needs: (1) places of refuge away from an unsafe partner in their new country [38] and (2) counseling to respond to experiences of sexual violence and attendant mental health consequences, such as suicidal ideation, PTSD, and anxiety [42,43].

Discrimination and Oppression. Women experienced discrimination and oppression [37,38,39] related to multiple and intersecting levels of oppression due to race, gender, immigration status, age, and language abilities [39] and to a lack of cultural, social, economic, and political capital [37]. Refugee women described suffering discrimination due to language barriers when accessing services and feeling like they had to exert more effort to access services, while also having fewer opportunities [38]. Challenges to accessing reproductive care were amplified by feelings of prejudicial attitudes and a lack of understanding from the service providers, as well as difficulties with being vulnerable and trusting any authority after the trauma they endured. Additionally, some of the intransigence and bureaucracy of the legal and other social structures themselves made it difficult for providers to give the care they wanted to the refugees [40].

#### 3.1.2. Structural Barriers to SRH Care

Precarity of Legal Status. Four studies noted the precarity of legal status that refugee women navigate while resettling in a host country [37,38,39,40]. Legal status was tied to access to health care, employment opportunities, and education options for children, such as subsidized daycare [38]. The uncertainty of documentation status and the looming possibilities of detention or deportation of themselves or loved ones created a “constant state of flux” [40] (p. 137), producing uncertainty, anxiety, and fear [37]. Unstable legal status and risks of removal compound with navigating heightened pregnancy stressors. For some women, precarious legal status was another tool of coercion and abuse. Precarious legal status also made it more difficult for women to access IPV and SRH services or government support for financial assistance [39].

Societal and Community Norms. Studies identified how societal and community norms influenced women’s experiences of seeking reproductive healthcare in resettlement. Public discourse and social norms about what constitute “violence” and “normal” relationship behavior influenced survivors’ perceptions of violence and their own experiences [41]. In the context of resettlement, women concurrently negotiated changing identities as they adjusted to new cultural and economic roles, sometimes leading to “cross-cultural clashes” [37] (p. d74). Family or kin living in the same country as resettled women often served as community and social support but also reinforced beliefs, values, and relationship expectations about gender, sex, and reproduction contrary to the resettlement country’s norms, sometimes producing stigma in women navigating sexual violence, pregnancy, or abortion after rape [37,39]. These pressures from family and society informed women’s decision-making about their health. Concurrently, there was sometimes a disconnect between what was viewed as cultural tradition by refugees, such as female genital cutting (FGC), and how their providers perceived these experiences as traumatic. This contradiction and conflict is supported by previous work on migrant women’s experiences with FGC and service providers perspectives on the tradition [44]. Thus, provider bias may impact the provision of SRH care [41].

Lack of Trust with Service Providers. Five studies [38,40,41,42,45] addressed relationships with service providers, organizations, and institutions, explicating how trust facilitated refugee women’s access to care or how a lack of trust hampered access to care. Prior negative experiences with organizations, both in their home country and their country of resettlement, undermined trust-building for service provision in resettlement contexts [38,40,42]. Distrust stems from organizations’ complicity in women’s migration trauma, personnel prejudice, and disdain for medical professionals [40]. This distrust also extended to other positions of authority. For example, some refugee women were victims of police violence, exploitative employers [38,42], or suspected that service providers were connected to authority figures who might be a threat [41]. This distrust led to women delaying or avoiding needed care, negatively impacting their wellbeing [41]. Conversely, in relationships where trust was established, refugee women were able to openly talk about experiences of sexual violence and receive medical and social support [40,45]. This trust was built through confidentiality, individual care, making refugee women feel heard and seen, and maintaining a good reputation [40,41].

#### 3.1.3. Actions, Practices, and Resources

Addressing Gaps in Information and Access to Care. In five articles [38,39,40,41,45], survivors were hampered by information and access gaps in obtaining care. They expressed limited knowledge about “when and where to access care,” [40] (p. 137) as well as the “nature” of care and support and “available societal resources” [41] (p. 6) for new mothers in the country of resettlement. Women were fearful and discouraged when trying to access vital care [38], limiting their use of services.

Providing Culturally Competent Services. Five articles [37,38,40,41] identified the need for culturally competent services. Service providers identified the value of attending to the cultural and ethnic backgrounds of immigrant women while avoiding cultural generalizations to provide care that is individualized for each refugee, immigrant, or asylee woman. Whether framed as cultural competence, cultural sensitivity, cultural tailoring, or multiculturalism, service providers emphasized the need for “taking into account the changes associated with the migration process, as much before and during migration as during resettlement” [38] (p. 423).

Providing Language Interpretation. Six studies referenced the need for interpretation services [37,38,39,40,41,43]. Language barriers were prevalent. Longer appointments with service providers to accommodate language-related needs were not always provided. Furthermore, language barriers led to miscommunication and confusion for patients and providers alike and compounded existing barriers to care. Language barriers also accentuated information and access gaps. To bridge language barriers and improve service delivery, providers working with women described the utility of investing in trained interpreters [41,43] and technology, for example, pre-recording messages for use in cases of emergency with no translator present [40].

Linking to Counseling and Emotional Support. Four articles [41,42,43,45] noted survivors’ needs for counseling and emotional support. These needs were met by people willing to provide “solidarity and prayer” [45] (p. ii), therapists specializing in psychosexual counseling [43], and the included visitation from unpaid volunteers for new mothers, which affirmed “the sense that they had an individual social value” [45] (p. ii). The key to successful support through these roles was active listening, unconditional acceptance, non-judgmental affirmation, perception-focused coping, moral support, and the ability to understand how histories of trauma and violence from migration intersected with survivors’ continuing stressors, such as legal status [45]. This suggests the possible benefits of increased services from trained nurses and social workers for refugees seeking SRH support.

Promoting Social Support. Finally, the need for social support surfaced as an important sub-theme in six of the studies [37,38,40,41,42,45]. Dimensions of social support included familial support: “there is no mum here, an asylum seeker does not have a mum” [40] (p. 5). The absence of social support left women isolated, lonely, and fully dependent on others, with only a few people that they could rely on. Isolation was compounded by linguistic barriers, homelessness, short-term housing necessitating frequent moves, or the need to stay home all day with children, truncating organic social support development. While some refugees had an informal support system, and some had family or kin living in the same country that provided social support [37,39], both patients and providers raised concerns that refugee women’s social support was insufficient, particularly for new arrivals “uprooted” from their social support systems in their home country [37] (p. e74).

### 3.2. Aim 2: Principles of Trauma-Informed Care Operationalized in Sexual and Reproductive Health Care for Survivors of IPV/Sexual Violence

#### 3.2.1. Empowerment

The principle of empowerment in sexual and reproductive health interventions, services, and programs was discussed in 16 articles. Empowerment encompassed education and skill-building among patients and organization members [46,47,48,49,50,51,52,53,54,55]. For example, providing patients with modules on health-related (e.g., nutrition, exercise, safe sex practices, HIV, relaxation) and educational content (parenting, budgeting) and ways to practice skills [52,56,57], or training with opportunities for role-play among providers (e.g., parent-infant relationship, taking a trauma history) [49,50,53,55]. Additionally, discussions of empowerment included support for staff through consistent trauma debriefing and clinical supervision [56,58]. The pre-existing presence of skilled, trained providers already within the organization’s infrastructure [59], as well as organizations already committed to and engaging in support for practitioners responding to client needs [47], facilitated efforts toward TIC.

The process of universally sharing (providers) and receiving information (patients) was deemed empowering, rewarding, and increased patients’ and providers’ awareness of IPV and support programs, which was identified as highly valuable [47,48,53]. Further, increasing access among patients and organization members to local, national, and government social and medical services and resources (e.g., violence-related, legal aid, substance use, trauma service providers, dental care, and gynecology) was identified as an essential component of empowerment [46,48,50,53,56,57,58,60,61]. Mechanisms of increasing access included staff from healthcare centers meeting and developing relationships with staff from local IPV organizations to facilitate and enhance comfort when providing patients with warm referrals, which was deemed gratifying [47,48,53]. Lastly, non-collusive practices (i.e., patient voice, choice, and control) and power sharing (e.g., therapeutic alliances) were seen as critical priorities [46,47,49,51,52,53,60]. This included thoroughly explaining procedures and asking permission to examine and touch a patient [59], following the pace of the patient and maintaining their autonomy and self-determination (e.g., acquiring birth control, vaccinations, vaginal exam), not being “…questioned excessively…or told what they could or could not do” [51] (p. 281), and providing patience with opportunities to “speak freely about their lives” [57] (p. 7).

Organizations also seemed aware of and committed to addressing power dynamics to create and facilitate their durable TIC intervention [46,50,58]. Power sharing, an important facilitator of empowerment, is related to greater client autonomy over their care and increased client choices in program delivery [51]. A commitment to addressing power dynamics includes attunement to linguistic and culturally specific needs [49,60], an aspect of TISRH services particularly relevant to refugee women. Reducing power imbalances inherent in medical care also took the form of greater client autonomy over program development, increased client choices, and supporting staff to be “present with clients across the dimensions of their lives” [46,50,51,58] (p. e59).

#### 3.2.2. Trauma-Specific Services and Integrated Care

The principle of trauma-specific services and integrated care within sexual and reproductive health interventions, services, and programs was discussed in 15 articles. The principle of trauma-specific services and integrated care was conceptualized as education on trauma-related symptomatology, including adaptive coping (e.g., dissociation, substance use) and methods of managing trauma-related symptoms, as well as integrating trauma-specific psychoeducational content and skill practice into programming [46,49,56,57,62,63]. Enacting this principle included providing therapeutic support, such as trauma-specific services, in which the goal is to address the effects of trauma, including methods for safe coping and strategies for change (e.g., emotion-focused coping, exercise, mindfulness), and managing triggers and feelings (e.g., shame, embarrassment, and to “feel nothing” or “forget” trauma) [56] (p. 2000) [46,54,62]. Organizational staff also considered the trauma histories of patients and the potential impact, including coping mechanisms, trauma may have on their present experiences and functioning [46,58]. This includes universal screening to prevent stigmatizing or profiling clients by identifying them as eligible for specific services [47] and offering services to everyone, whether they were screened in or not [53]. Additionally, prevention of vicarious trauma among staff was discussed [55,56], including the need for psychological counselors to provide routine trauma debriefing as a means of preventing burnout among staff [56].

Further, articles discussed that trauma-informed integration includes mental health screening and readily available on-site services (e.g., therapeutic and psychiatric support) within organizations and clinics, such as medical homes, for patients [52,60], which allowed for effective and timely coordinated care [61]. Implementation of this principle included the integration of interventions, services, and programming within local “host” organizations and co-locations [49] (p. 544), such as integrating a medical home within a pediatric hospital [60], as well as home visits that supported clients in feeling cared for and supported [56]. Another key element of TIC included providing patients and clients with, or referring to, comprehensive services (e.g., case management, interpreters, substance use treatment, dental care, podiatry, gynecology, therapeutic support, childcare, and transportation) provided by a multidisciplinary team [51,52,58,60,61]. Connecting patients to community services (e.g., IPV victim advocate) in real-time [47,53] and coordinating care across multiple institutional settings (e.g., psychiatric hospital, jail, shelter) [58] were essential in implementation. System-level coordination, which was a key facilitator in creating trauma-specific services and integrated care, is characterized as reducing contact with multiple systems by providing a variety of coordinated, integrated, interdisciplinary services in one place and is a key aspect in promoting sustained engagement for survivors and program sustainability [49,51,60,61]. System-level coordination included a commitment to program sustainability and providing time and resources for effective implementation [47,53].

#### 3.2.3. Connection

The principle of connection was highlighted in 14 articles. Connection was seen as fostering alliances between the intervention or program personnel and the community (e.g., city health officials) and the population in which they serve [46,48], as well as between organization members and patients [49,51,52,57,60]. This included intensive case management between staff and service recipients, in which barriers to accessing community services and engagement in care, along with providing supported referrals, were addressed [46,57,58]. Providing universal IPV education and screening was seen as a connection point that signified provider care and concern for patients’ well-being, while learning about violence-related resources was a connection point for patients to share with people in their network [47,53]. Connection was also described as fostering alliances through mutual peer support and mentoring among patients and clients (e.g., delivery of interventions within a group setting, community peer support networks, baby shower) to create a sense of community, partnership, and support for change [46,51,52,56], with similar sentiments regarding connections among providers and staff (e.g., weekly case conferences to assess patient progress and strategize support) [58]. However, questions of emotional safety within a group format arose, including hesitation to open up and worrying others in the group would judge them [56,57].

Mechanisms of facilitating connection included the importance of early engagement in “setting the tone”, time to connect and bond, feeling “listened to and heard”, and receiving consistent support [51] (p. 281), which were essential in building trust and rapport among practitioners and clients [49,51,61]. Examples of connection were sharing in a communal meal or engaging in other programmatic activities together, such as attending a health education training, waiting for appointments, or attending a court hearing [52,58]. Additionally, connection was portrayed as assessing for, supporting, and facilitating opportunities to nurture patients’ and clients’ familial, social, and community relationships (e.g., intimate partners) [49,59]. For example, sharing information and providing resources (e.g., smartphones and data) and services (e.g., clinical care) to patients’ family members, friends, and others in their support network (e.g., program staff) [49,50,51,52,53].

#### 3.2.4. Safety

The principle of safety in SRH interventions, services, and programs was discussed in 14 articles. This principle was described as emotional and physical safety and meeting basic needs. Emotional safety encompasses non-judgmental, non-assumptive, sensitive, and empathetic alliances and dialogue between staff and clients, including when discussing histories of violence [47,48,49,55,58,60]. This included the use of motivational interviewing techniques (e.g., reflective listening, affirmations) [46,59]. Further, participants emphasized the importance of feeling welcomed, valued, and cared for [51,57], and facilitators of a didactic module and role-play for obstetrics and gynecology residents reported utilizing “…clear, non-judgmental, and empathic” responses to patients [55] (p. 5). Emotional safety included reducing redundant trauma disclosure, such as obtaining only pertinent information to make informed decisions and circumscribing roles [46,52,60], and recognizing that, for some people, pregnancy may be a trauma [46]. Further, a key aspect of emotional safety included universal education and screening for violence in relation to SRH outcomes irrespective of trauma disclosure [47,48,50,53], which helped to avoid “alienating someone…or profiling them” [47] (p. 962). Emotional safety was also described as maintaining the confidentiality of patient information by providing ancillary staff with information only if relative to one’s care [52,58], and offering alternative options for notifying abusive partners of healthcare results (e.g., STI) [50].

Physical safety encompassed the creation of a welcoming, calm, and non-judgmental physical atmosphere [47,52], including appealing, comprehensive, and accessible intervention and program materials (e.g., simplified language, accessible to multiple educational levels) [56]. Descriptions of physical safety also included geographic and financial accessibility [52,58,60] and privacy [52,59], as well as the presence of security officers within the clinic [46,52]. Safety was also discussed in relation to meeting the basic needs of patients and clients, including confirming or acquiring safe housing or emergency shelter, food, clothing, toiletries, employment, transportation (e.g., alternative to public transit), and legal and social services (e.g., communication of restraining orders) [46,51,52,58,59,60]. A “safe space,” including a space free from judgmental messaging from peers or professionals, in and of itself was a critical facilitator in participants’ feelings of safety, as was genuine care from providers, which enhanced their willingness and comfort to receive services [47,48]. The papers cited in this section did not discuss challenges when screening for violence across languages and cultures.

#### 3.2.5. Collaboration

The principle of collaboration in SRH interventions, services, and programs was emphasized in 11 articles. Collaboration was described as multisectoral stakeholder engagement, including potential and current service users, content experts, service providers within local programs, government officials and community leaders, and community advisory boards. Stakeholder involvement encompasses various capacities, including feedback on the intervention and program design and development, processes, and content (e.g., revising marketing material), as well as evaluation and interpretation of intervention findings [48,49,50,56,57]. Multisectoral collaboration assists stakeholders in jointly defining the problem and intervention targets and developing a shared understanding of how to resolve the problem [48]. Further, communication and responsive programming to service users’ needs acted as facilitators of multi-sectoral collaboration, resulting in effective implementation of TISRH services and programs, and for which implementation at the beginning stage generates support and buy-in to develop sustainable programs that are more likely to be endorsed by the larger community [48,57].

Collaboration also encompassed survivor-and-patient-centered practices, described as “meeting patients where they are” [53] (p. 86), both emotionally (e.g., allowing patients to disclose when they were ready) and physically (e.g., physicians meeting clients in various settings) [62] and being available on clients’ time [51]. Further, collaboration was discussed as multi-stakeholder cooperation (e.g., physician and social worker) in discussing patients’ histories prior to engaging the patient to reduce retraumatization resulting from repetitive trauma-related screening among patients [52,59]. Collaboration was also portrayed as leveling power dynamics among staff and patients, such as joint goal setting [59], sharing meals [46,58], coordinating services among a client’s family unit (e.g., mother and father) [49], and continuous dialogue between stakeholders, particularly service providers and service users [48,57]. Further, when providers are responsive to service user requests and suggestions, a feedback loop is created wherein users can communicate barriers to their involvement and providers can address them [57], thus strengthening the foundation for trusting, collaborative relationships necessary to facilitate service delivery [51,57]. Responsive programming also includes a commitment to providing time, resources, and flexibility as a means of effective implementation and sustained programmatic engagement [46,48,53]. Flexibility includes modifying interventions to meet clients’ needs and ease the burden of use, such as shortening the number and length of intervention sessions, having flexible hours to meet client scheduling needs, having no penalties for late or missed appointments, and considering clients’ needs within the broader context of their relationships, families, and communities [46,49]. For example, collaboration included focusing on survivors’ families, such as childcare, or bringing the services to geographic locations where clients would find them safe, accessible, and convenient to support client access to care and retention [46,48,51].

#### 3.2.6. Identity, Culture, and Context

Principles related to identity, culture, and context in SRH interventions, services, and programs were discussed in 12 articles. Aspects of identity, culture, and context among patients were described as minimizing and working to avoid labels, stigmas, and stereotypes [46,49,55], including around IPV and sexual violence victimization. Among organizational members, identity, culture, and context were portrayed as education, training, and enhanced awareness and empathy of the widespread prevalence of traumatization, along with the signs, symptoms, and impact of IPV, SV, homelessness, and HIV diagnosis on women’s SRH outcomes (e.g., pregnancy) and decision-making behavior, including choices related to clinical recommendations, [50,53,55,58,59,62]. Further, a critical mechanism of this principle was education on how trauma exposure may influence a patient’s present behavior (e.g., health decision), such as their service-seeking and service engagement [52,58] including the connection between violence, gender, racism, poverty, and trauma [46], as well as integrating this information into an organization’s policies and practices to reduce re(traumatization) [53]. Similarly, patients described the receipt of IPV education and resource materials as thought-provoking and said that it too raised their awareness [47].

Further, awareness of the structural and bureaucratic vulnerabilities clients face (e.g., trouble navigating the healthcare system, transportation) and providers and staff encounter (e.g., lack of personnel and resources) was key in TI implementation, along with taking action to support clients, such as physically waiting alongside patients when seeking resources from state agencies (e.g., food stamps) [58,61]. Identity, culture, and context also included provider training on delivering quality, culturally appropriate care based on the cultural, linguistic, and medical considerations and needs of the populations they serve [52,57]. For instance, hiring bilingual staff throughout an organization, including providers, nurses, and medical assistants [52], and considering providers’ gender [46].

#### 3.2.7. Trustworthiness

The principle of trustworthiness in SRH interventions, services, and programs was discussed in seven articles. The principle of trustworthiness was conceptualized as developing trust and rapport through clear, thorough explanations of the intervention, program, or services and the use of information, along with the option for program recipients to ask questions and to be informed of potential discomfort associated with trauma screening [46,51,59]. Trustworthiness, in relation to safety, was also discussed as continuity of care, such as patients seeing the same provider and the provider offering a warm handoff [46,52,60], consistency of staff presence through outreach [58], as well as reliability, described as an “…unwavering, ever-present” support from staff [51] (p. 281). A mechanism of trust and safety was also clear communication; for example, providers communicated transitions of care [46]. Receiving support and developing trust with staff served as a critical aspect in selecting birth control, including addressing fear related to the client’s reproductive health, safety, and well-being [51]. The words of one individual to their social worker, who provided intensive HIV case management, “…you did not give up on me, never”, exemplify the aspect of consistency in relation to trust [58] (p. e56). Concerns for providers losing patients’ trust included the potential for patient feelings of betrayal in instances of mandatory reporting of IPV related to possible child abuse [47].

## 4. Discussion

The findings from this scoping review highlight significant SRH needs among resettled refugee women who have experienced IPV and/or sexual violence and the ways in which principles of trauma- and violence-informed care have been put into practice to address the SRH of women in resettlement contexts.

In relation to the first study aim, the analysis emphasized the centrality of violence and trauma in refugee women’s lives and migration trajectories, expressed through power and control over their bodies, histories of sexual violence, and experiences of discrimination and oppression. Precarity of legal statuses, societal and community norms, and lack of trust of formal service providers form structural barriers to SHR care that are difficult for women to overcome [37,38,39,40,41,42,45]. To address these needs and challenges, practitioners have sought to address gaps in information and access to care, focus on providing culturally component services, ensure access to language interpretation, connect women to counseling and emotional support, and promote social support [38,39,40,41,43,45]. Service providers may not understand the complexity of challenges in the migration journey or the physical and psychosocial implications of sexual violence. It is important that social service organizations offer educational opportunities to practitioners to build knowledge and skills on these topics as part of organizational-level efforts to institutionalize high-quality, accessible trauma-informed care. These findings emphasize the imperative for integrating trauma-informed care into the design and delivery of SRH for women in refugee resettlement.

For the second study aim, the analysis highlighted efforts to integrate and operationalize principles of trauma-informed care in SRH care for women in resettlement in recognition of the multiple and intersecting experiences of violence they may have experienced, including but not limited to IPV and sexual violence. Prior research recommends providing trauma-informed SRH care to better support IPV and sexual violence survivors, particularly those who experienced unwanted pregnancies and miscarriages as a result of abuse and/or coercion [40,41,45]. The studies highlight the importance of integrating principles of safety, trustworthiness, identity, culture, and context to create welcoming and comforting service environments that will act as a refuge from violence [38], in which women feel physically and emotionally safe. The trauma-informed principles of connection and collaboration appeared particularly vital in the provision of SRH care to refugee women, including between service providers and clients [40,41,45], and organizations and refugee communities [38,40,42,45]. These connections and collaborations at multiple levels can address vulnerabilities, barriers, and gaps identified in the first aim by systematically building trust, addressing gaps in information and in services, and building a community of support.

With the emphasis of particular trauma-informed care principles (e.g., trauma-specific services and integrated care, connection) over others (e.g., collaboration; identity, culture, and context), the question arises as to whether all, or only some, of the principles need to be implemented in the design and delivery of services to indeed be a TIC intervention, program, and practice. What constitutes robust and high-quality trauma-informed SHR care? The findings highlight the need to develop a comprehensive framework of trauma-informed SRH care for refugee women in resettlement. Bringing TIC principles to bear is an organizational undertaking [33,34,35] and the current analysis raises another question: can an existing organization or program be retroactively “re-organized” or “re-programmed” to successfully provide trauma-informed SRH care? In other words, can a trauma-informed approach be embedded after the fact, or does it need to be built from the ground up, with an attunement to trauma baked into its very foundation? Barriers to trauma-informed SRH care noted across the studies in this review indicate that it is difficult to shift an organization toward a trauma-informed approach when its institutional structure may perpetuate a lack of safety, deny user choice, be bereft of collaboration and trustworthiness, and fundamentally erode empowerment [38,40,42]. Indeed, trauma-informed care must inform every aspect of an organization; it must be evident in the mission and the allocation of resources [64,65]. While not explicitly noted in the reviewed studies, legal and social structures in resettlement countries may also be a barrier to effective TIC care or exacerbate the traumatic impacts of sexual violence through recent legislation limiting reproductive autonomy [66,67].

The costs of developing and institutionalizing trauma-informed SRH care may seem prohibitive [64]. Committing to and actualizing the tenets of trauma-informed care requires qualified and trained staff, ongoing staff development, consistent engagement with community stakeholders, facility creation and upkeep, and financial resources to support trauma-informed organizational practice and facilitate access to services [64,65,68]. The limited literature identified in our search may reflect the dearth of funding available to develop and sustain trauma-informed SRH care for women in resettlement.

It is important to note that none of the studies included in the current analysis evaluated the effectiveness of trauma-informed SRH services for refugee women in a resettlement context. These findings point to the need for rigorous evaluation and methods to assess the implementation of trauma-informed principles, their impact on staff and patient outcomes, and whether the latter is impacted by the former. Future research should also measure and track a cost analysis of implementing trauma-informed SRH care for refugee patients over time. While there may be significant up-front costs to set up and maintain a clinic that adheres to trauma-informed principles, in the long run, this value-based care may save money for both health service providers and their patients [69,70].

The Refugee Women’s Health Clinic (RWHC), launched in 2008 at Valleywise Health in Phoenix, Arizona, is an exemplar of healthcare providers, allied professionals, and community stakeholders working in collaboration to address the SRH needs of refugee women from over 71 countries. Steeped in principles of trauma-informed care and health equity, the RWHC is a nationally recognized model of patient-centered care [71]. Attunement to historical, cultural, and gender disenfranchisement was the impetus for the clinic’s creation. The RWHC was intentionally built with the goal of providing trauma-informed care, and the clinic defines, implements, and measures each trauma-informed care principle [72].

Key clinic challenges center around the stigmatization of SRH and deep distrust of health services due to historic and ongoing medical racism. Clinic founders are aware of the medical field’s legacy of neglect, discrimination, and violence towards women of color and developed the clinic in close collaboration with local ethnic communities as the “eyes and ears” to identify health needs and build a program in direct response to meet community-voiced needs. Recognizing the community as experts and ongoing engagement with community partners and clients has helped the clinic identify missed-steps and take accountability for course-correction. The RWHC depends on an interdisciplinary advisory coalition with more than 60 stakeholders, including ethnic organizations, faith-based community agencies, the public health department, resettlement agencies, mental health service providers, and academic partners, to offer comprehensive SRH services that are culturally attuned, linguistically informed, and proximal and accessible to refugee women.

Clinicians and staff at the RWHC promote the importance of maintaining a culture of safety by creating a space where women and their families feel heard. A team of cultural health navigators (CHNs) serves as the lifeline between refugee communities and complex health systems, helping socio-ethno-linguistically diverse refugee patients navigate complex systems of care and fostering trust [73]. CHNs are the foundation of the RWHC, possessing lived experience of forced displacement, and comprise a multi-lingual staff speaking over 20 languages fluently as certified medical interpreters. They facilitate an integrated team-based approach to health care delivery that involves intensive care coordination and case management that engenders trust and empowers women towards enhanced health literacy, self-efficacy in navigating the health care system, and optimizing their reproductive and sexual health and well-being. Emphasizing medical literacy and effective communication empowers service users to make decisions for their own health, have access to improve their own quality of life and wellbeing for themselves and their families, and help center client dignity and autonomy. Having someone just a phone call away who understands their story and can meet them where they are reduces retraumatization and stigmatization and helps synchronize family care by connecting patients to other services and supporting them through warm hand-offs and follow-up to make sure that the patient does not feel like they are on their own.

The clinic offers comprehensive SRH services, including obstetric, gynecologic, preventative health care, and family planning, with specialties in reconstructive procedures and surgical defibulation for women who have experienced FGM/C [74,75,76,77]. Efforts to rigorously evaluate the impact of the RWHC on patient outcomes are currently underway.

### Limitations

The study is limited by its time period of research; articles were only included within a specific publication date range. The impetus for this study has fueled other practitioners and researchers’ increased attention towards the health and wellbeing of migrating people and the necessity of TIC, producing new publications in this area [78]. It is important to note that all of the articles predate the COVID-19 pandemic, which had a significant impact on all healthcare systems around the world and shaped resources and access to reproductive healthcare for immigrant women [79,80]. This scoping review was further limited to articles published in English due to the language skills of those on our team; however, we cannot ignore the exclusionary implications of doing so, particularly in an area of research and practice that centers people representing extraordinarily socio-ethno-linguistic diversity. The reference lists from the included articles were not examined as part of screening articles for the scoping review; only papers collected through the six databases were included to keep the searches uniform and replicable. Our exclusive focus on scholarly literature similarly limits our analysis.

## 5. Conclusions

Recent events and rulings in resettlement countries, including the United States, remind us that we cannot take for granted the availability of SRH services and reiterate the importance of front-line organizations committing to ensuring and sustaining access to trauma-informed SHR care for all women, and particularly those migrating in search of safety; this is an important avenue for future research. However, the landscape of TIC-SRH services for migrating women remains bleak. These studies together indicate a roadmap to improve services for survivors of gender violence and increase health and wellbeing outcomes for migrating women.

## Figures and Tables

**Table 1:** Search Terms.

Concept	Associated Search Terms
Intimate partner and sexual violence	“sexual violence” OR “rape” OR “sexual assault” OR “sexual coercion” OR “reproductive coercion” OR “gender based violence” OR “gender-based violence” OR “GBV” OR “intimate partner violence” OR “IPV” OR “abuse” OR “domestic violence” OR “DV” OR “violence against women” OR “VAW” OR “sexual abuse” OR “sexual trauma”
2. Sexual and reproductive health	“sexual health” OR “reproductive health” OR “perinatal” OR “postnatal” OR “obstetric*” OR “pregnan*” OR “miscarriage” OR “sexually transmitted infection” OR “sexually transmitted disease” OR “STD” OR “STI” OR “HIV” OR “AIDS” OR “HIV/AIDS” OR “contraception” OR “fertility” OR “maternal morbidity” OR “fistula” OR ((“unwanted” or “unplanned” or “forced” or “rape-related”) AND “pregnan*”) OR “endometriosis” OR “vaginal bleeding” OR “cervical cancer” OR “HPV” OR “ovarian cancer”
3. Refugee	“refugee*” OR “asyl*” OR “forced migra*” OR “migra*”
4. Trauma-informed care	“Trauma-informed” OR “Trauma informed” OR “Trauma-specific” OR “trauma specific” OR “Trauma-integrated” OR “Trauma-integrated” OR “Trauma-based” or “Trauma based”) AND (“Care” OR “Practice*” OR “Response*” OR “Service*” OR “Approach*” OR “Organization*” OR “Treatment*” OR “System*”

**Table 2:** Overview of Findings.

**Aim 1: Factors shaping SRH needs and access to SRH care among refugee women in resettlement who have experienced IPV and/or sexual violence**	
*Themes*	*Subthemes*
Centrality of violence and trauma	Power and control in reproduction and bodily autonomy History of sexual violence Discrimination and oppression
Structural barriers to SRH care	Precarity of legal statuses Societal and community norms Lacking trust with service providers
Actions, practices, and resources	Addressing gaps in information and access to care Providing culturally competent services Providing language interpretation Linking to counseling and emotional support Promoting social support
**Aim 2: Principles of trauma-informed care operationalized in sexual and reproductive health care for refugee women in resettlement**	
*Principles*	*Examples*
Empowerment	Validate and prioritize patients and organizational members options, voice (e.g., storytelling), choice, control (e.g., shape the focus of work and influence program services), and resilience (e.g., belief in healing and recovery) Build off patients and organizational members strengths and skills (e.g., social roles, opportunities for leadership development) and expand training, access to resources, and support network
Trauma- specific services and integrated care	Acknowledge that trauma-related symptoms originate from strategies to adapt to and cope with traumatic events(s) or context Organization provides trauma-specific services (e.g., cognitive behavioral therapy, EMDR) within programming aimed at addressing the effects of physical and sexual violence and emotional abuse and strategies for coping or refers to those services Organization reflects a holistic culture of healing and prioritizes women’s mental, physical, sexual, and spiritual health
Connection	Prioritize and facilitate opportunities for mutual peer support among patients Organizational members invest in relationships with patients Support patients’ parental, social, and community relationships
Safety	Physical safety of patients and organizational members, such as setting, environment, welcoming, minimizing triggers, supportive for disclosure, and materials that are inclusive and culturally appropriate Emotional safety of patients and organizational members, such as being non-judgmental, respectful, adhering to confidentiality, not repeatedly collecting violence/trauma-related questions, and obtaining only necessary information Basic needs (e.g., safe housing, shelter, food, healthcare)
Collaboration	Maximize respect, collaboration, mutuality, and shared power (e.g., decision-making) between patients and organizational members (e.g., patients seen as experts on their own health and well-being, mutual goal setting) Leveling of power differentials (e.g., changing paternalistic practices, organizational makeup) Continuous solicit of patient, consumer, and service recipient involvement and input in organizational design and evaluation (e.g., services)
Identity, culture, & context	Addresses stigma, cultural stereotypes, and biases embedded in organization Inclusive, welcoming, and affirming (e.g., physical space) of all backgrounds and multiple identities (e.g., gender, sexual orientation, race, class, nationality, disability, religion, age) Prioritizes racial, ethnic, cultural, and gender considerations and needs (e.g., the organization leverages and provides access to gender-responsive services and traditional cultural connections) Organizational members are educated and responsive to the drivers and impacts of historical and present-day trauma and structural oppression, and their influence on lived experiences
Trustworthiness	Clear and transparent decision-making and information for patients and organizational members Consistent and predictable relationships, practices, and boundaries

**Table 3:** Articles by Aims, Research Population, and Themes.

Article	Research Population	Themes
**Aim 1—Factors that shape SRH needs and access to SRH care**		
Balaam et al., 2015	Refugee or asylum-seeking women in the United Kingdom (UK)	History of Sexual Violence; Precarity of Legal Status; Lack of Trust with Service Providers; Addressing Gaps in Information and Access to Care; Providing Culturally Competent Services; Providing Language Interpretation; Promoting Social Support
Byrskog et al., 2015	Somali-born refugee women in Sweden	History of Sexual Violence; Societal and Community Norms; Lack of Trust with Service Providers; Addressing Gaps in Information and Access to Care; Providing Culturally Competent Services; Providing Language Interpretation; Linking to Counseling and Emotional Support; Promoting Social Support
Mantovani 2013	Mostly migrant and asylumseeking mothers from Africa, in the UK	Power and Control in Reproduction and Bodily Autonomy; History of Sexual Violence; Discrimination and Oppression; Precarity of Legal Status; Societal and Community Norms; Providing Culturally Competent Services; Providing Language Interpretation; Promoting Social Support
McLeish & Redshaw 2021	Mostly immigrant, refugee, and asylum-seeking mothers in the UK	Lack of Trust with Service Providers; Addressing Gaps in Information and Access to Care; Linking to Counseling and Emotional Support; Promoting Social Support
Mehta & Gagnon 2016	Refugee and asylum-seeking women from Africa, Asia, and Latin America in Canada	History of Sexual Violence; Lack of Trust with Service Providers; Linking to Counseling and Emotional Support; Promoting Social Support
Ochoa & Sampalis 2014	Latin-American migrant women in Canada	Power and Control in Reproduction and Bodily Autonomy; History of Sexual Violence; Discrimination and Oppression; Precarity of Legal Status; Lack of Trust with Service Providers; Addressing Gaps in Information and Access to Care; Providing Culturally Competent Services; Providing Language Interpretation; Promoting Social Support
Rogstad & Dale 2004	Asylum-seeking women in the UK	History of Sexual Violence; Providing Language Interpretation; Linking to Counseling and Emotional Support
Tarzia et al., 2021	Providers for minority women’s legal and domestic service needs in Australia	Power and Control in Reproduction and Bodily Autonomy; History of Sexual Violence; Discrimination and Oppression; Precarity of Legal Status; Societal and Community Norms; Addressing Gaps in Information and Access to Care; Providing Language Interpretation
**Aim 2—TIC principles and SHR care**		
Ashby et al., 2019	Pregnant and parenting adolescent girls in the United States (US)	Empowerment; Trauma-specific Services and Integrated Care; Connection; Safety; Collaboration; Identity, Culture, and Context; Trustworthiness
Decker et al., 2017	Women receiving family planning care in the US	Empowerment; Trauma-specific Services and Integrated Care; Connection; Safety; Identity, Culture, and Context, Trustworthiness
Decker et al., 2018	Female sex workers receiving HIV care in the US	Empowerment; Connection; Safety; Collaboration
Domoney et al., 2019	Women receiving domestic violence and parenting services in the UK	Empowerment; Trauma-specific Services and Integrated Care; Connection; Safety; Collaboration; Identity, Culture, and Context
Hill et al., 2019	Women receiving sexual and reproductive health care in the US	Empowerment; Connection; Safety; Collaboration; Identity, Culture, and Context
Kachingwe et al., 2019	Homeless adolescent girls receiving holistic sexual health care in the US	Empowerment; Trauma-specific Services and Integrated Care; Connection; Safety; Collaboration; Trustworthiness
Kalokhe et al., 2020	Providers and staff at an urban HIV care center in the US	Empowerment; Connection; Safety; Collaboration; Identity, Culture, and Context; Trustworthiness
Kappel et al., 2020	Providers at a clinic for sex trafficked youth in the US	Empowerment; Trauma-specific Services and Integrated Care; Connection; Safety; Identity, Culture, and Context; Trustworthiness
McNiel et al., 2014	Providers at an interdisciplinary medical home for survivors of trafficking in the US	Empowerment; Trauma-specific Services and Integrated Care; Connection; Safety; Collaboration; Identity, Culture, and Context; Trustworthiness
Miller et al., 2017	Providers and patients at family planning clinics in the US	Empowerment; Trauma-specific Services and Integrated Care; Connection; Safety; Collaboration; Identity, Culture, and Context
Myers et al., 2018	Trauma-exposed young women who use substances in South Africa	Empowerment; Trauma-specific Services and Integrated Care; Connection; Safety, Collaboration
Myers et al., 2019	Trauma-exposed young women who use substances in South Africa	Empowerment; Trauma-specific Services and Integrated Care; Connection; Safety; Collaboration
O’Conner et al., 2021	Women who used methamphetamines during pregnancy and post-partum in Australia	Trauma-specific Services and Integrated Care; Identity, Culture, and Context
Piper et al., 2020	Providers, staff, and patients at an HIV treatment center in the US	Empowerment; Trauma-specific Services and Integrated Care; Connection; Identity, Culture and Context
Powers et al., 2017	Criminal-legal system involved patients diagnosed with HIV, mental health issues, and substancerelated disorders in the US	Empowerment; Trauma-specific Services and Integrated Care; Connection; Safety; Collaboration; Identity, Culture, and Context; Trustworthiness
Rowe et al., 2014	Pregnant women with abuse-related post-traumatic stress in the US	Trauma-specific Services and Integrated Care
Salvador et al., 2020	Pregnant or parenting women admitted into a residential treatment program for substance abuse treatment in the US	Empowerment; Trauma-specific Services and Integrated Care
Stevens et al., 2020	Obstetrics service providers in the US	Empowerment; Trauma-specific Services and Integrated Care; Safety; Identity, Culture, and Context

## References

[1] Tan S.E., Kuschminder K. (2022). Migrant Experiences of Sexual And Gender-Based Violence: A Critical Interpretative Synthesis. Glob. Health.

[2] United Nations High Commissioner for Refugees [UNHCR] (2023). Global Trends: Forced Displacement in 2021. https://www.unhcr.org/en-us/publications/brochures/62a9d1494/global-trends-report-2021.html.

[3] UN High Commissioner for Refugees (UNHCR) (2018). Global Compact on Refugees. https://www.refworld.org/docid/63b43eaa4.html.

[4] Shahimi F., Block K., Alisic E. (2024). Sense of Identity Among Young People with Refugee Backgrounds: A Scoping Review. Child. Youth Serv. Rev..

[5] Freedman J., Freedman J., Kivilcim Z., Baklacıoğlu N.O. (2017). Women’s Experience of Forced Migration: Gender-Based Forms of Insecurity and the Uses of “Vulnerability”. A Gendered Approach to the Syrian Refugee Crisis.

[6] Cook Heffron L., Wachter K., Rubalcava Hernandez E. (2022). “Mi Corazón Se Partió En Dos”: Transnational Motherhood at The Intersection Of Migration And Violence. Int. J. Environ. Res. Public Health.

[7] Mouriki D., Lynch O., Windle J., Ahmed Y. (2021). Sexual and gender-based violence against refugee women as a continuum of violence. Giving Voice to Diversity in Criminological Research.

[8] Wachter K., Cook Heffron L., Devaney J., Bradbury-Jones C., Macy R., Øverlien C., Holt S. (2021). Intimate partner violence against women in forced migration. The Routledge Handbook of Domestic Violence and Abuse.

[9] Egli-Gany D., Aftab W., Hawkes S., Abu-Raddad L., Buse K., Rabbani F., Low N., Onarheim K. (2021). The Social and Structural Determinants Of Sexual And Reproductive Health And Rights In Migrants And Refugees: A Systematic Review Of Reviews. East. Mediterr. Health J..

[10] Wachter K., Dalpe J., Cook Heffron L. (2019). Conceptualizations of Domestic Violence–Related Needs Among Women Who Resettled To The United States As Refugees. Soc. Work. Res..

[11] Wachter K., Cook Heffron L., Dalpe J. (2022). “We Weren’t Ready”: Provider Perspectives on Addressing Intimate Partner Violence Among Refugees And Immigrants In The United States. J. Fam. Violence.

[12] Aguirre N.G., Milewski A.R., Shin J., Ottenheimer D. (2020). Gender-Based Violence Experienced by Women Seeking Asylum In The United State: A Lifetime Of Multiple Traumas Inflicted By Multiple Perpetrators. J. Forensic Leg. Med..

[13] Rees S.J., Fisher J.R., Steel Z., Mohsin M., Nadar N., Moussa B., Hassoun F., Yousif M., Krishna Y., Khalil B. (2019). Prevalence And Risk Factors Of Major Depressive Disorder Among Women At Public Antenatal Clinics From Refugee, Conflict-Affected, and Australian-Born Backgrounds. JAMA Netw. Open.

[14] Connor J.J., Hunt S., Finsaas M., Ciesinski A., Ahmed A., Robinson B. (2016). “Bean” E. Sexual Health Care, Sexual Behaviors And Functioning, And Female Genital Cutting: Perspectives From Somali Women Living In The United States. J. Sex Res..

[15] Metusela C., Ussher J., Perz J., Hawkey A., Morrow M., Narchal R., Estoesta J., Monteiro M. (2017). “In My Culture, We Don’t Know Anything About That”: Sexual and Reproductive Health Of Migrant And Refugee Women. Int. J. Behav. Med..

[16] Kisindja R.M., Kimona C., Etoy M., Dorme F., Benfield N. (2017). Family Planning Knowledge And Use Among Women In Camps For Internally Displaced People In The Democratic Republic Of The Congo. Int. J. Gynecol. Obstet..

[17] Ngum Chi Watts M.C., McMichael C., Liamputtong P. (2015). Factors Influencing Contraception Awareness and Use: The Experiences of Young African Australian Mothers. J. Refug. Stud..

[18] Jackson K.T., Mantler T. (2017). Examining the Impact of Posttraumatic Stress Disorder Related to Intimate Partner Violence on Antenatal, Intrapartum and Postpartum Women: A Scoping Review. J. Fam. Violence.

[19] Sperlich M., Seng J.S., Li Y., Taylor J., Bradbury-Jones C. (2017). Integrating Trauma-Informed Care Into Maternity Care Practice: Conceptual and Practical Issues. J. Midwifery Women’s Health.

[20] Ahmed A., Bowen A., Feng C.X. (2017). Maternal Depression in Syrian Refugee Women Recently Moved to Canada: A Preliminary Study. BMC Pregnancy Childbirth.

[21] Nakash O., Nagar M., Lurie I. (2016). The Association between Postnatal Depression, Acculturation and Mother-Infant Bond Among Eritrean Asylum Seekers in Israel. J. Immigr. Minor. Health.

[22] Tobin C.L., Di Napoli P., Beck C.T. (2018). Refugee and Immigrant Women’s Experience Of Postpartum Depression: A Meta-Synthesis. J. Transcult. Nurs. Off. J. Transcult. Nurs. Soc..

[23] Mohammadi S., Carlbom A., Taheripanah R., Essén B. (2017). Experiences of Inequitable Care Among Afghan Mothers Surviving Near-Miss Morbidity in Tehran, Iran: A Qualitative Interview Study. Int. J. Equity Health.

[24] Cuevas K.M., Balbo J., Duval K., Beverly E.A. (2018). Neurobiology of Sexual Assault And Osteopathic Considerations For Trauma-Informed Care And Practice. J. Osteopath. Med..

[25] Ottenheimer D., Huda Z., Yim E.T., Atkinson H.G. (2022). Physician Complicity in Human Rights Violations: Involuntary Sterilization Among Women from Mexico And Central America Seeking Asylum In The United States. J. Forensic Leg. Med..

[26] Scott J., Mullen C., Rouhani S., Kuwert P., Greiner A., Albutt K., Burkhardt G., Onyango M., VanRooyen M., Bartels S. (2017). A Qualitative Analysis of Psychosocial Outcomes Among Women with Sexual Violence-Related Pregnancies in Eastern Democratic Republic Of Congo. Int. J. Ment. Health Syst..

[27] Straehle C. (2019). Asylum, Refuge, And Justice in Health. Hastings Cent. Rep..

[28] Davidson N., Hammarberg K., Romero L., Fisher J. (2022). Access to Preventive Sexual and Reproductive Health Care for Women From Refugee-Like Backgrounds: A Systematic Review. BMC Public Health.

[29] Asif S., Baugh A., Jones N.W. (2015). The Obstetric Care of Asylum Seekers And Refugee Women In The UK. Obstet. Gynaecol..

[30] Harris M.E., Fallot R.D. (2001). Using Trauma Theory to Design Service Systems.

[31] Elliott D.E., Bjelajac P., Fallot R.D., Markoff L.S., Reed B.G. (2005). Trauma-Informed or Trauma-Denied: Principles and Implementation Of Trauma-Informed Services For Women. J. Community Psychol..

[32] Fallot R.D., Harris M. (2008). Trauma-Informed Approaches to Systems of Care. Trauma Psychol. Newsl..

[33] Hales T., Kusmaul N., Nochajski T. (2017). Exploring the Dimensionality Of Trauma-Informed Care: Implications For Theory And Practice. Hum. Serv. Organ. Manag. Leadersh. Gov..

[34] Substance Abuse and Mental Health Services Administration (2014). SAMHSA’s Concept of Trauma and Guidance for a Trauma-Informed Approach. https://store.samhsa.gov/sites/default/files/sma14-4884.pdf.

[35] Wilson J.M., Fauci J.E., Goodman L.A. (2015). Bringing Trauma-Informed Practice to Domestic Violence Programs: A Qualitative Analysis Of Current Approaches. Am. J. Orthopsychiatry.

[36] Colquhoun H.L., Levac D., O’Brien K.K., Straus S., Tricco A.C., Perrier L., Kastner M., Moher D. (2014). Scoping reviews: Time for clarity in definition, methods, and reporting. J. Clin. Epidemiol..

[37] Mantovani N., Thomas H. (2014). Choosing Motherhood: The Complexities of Pregnancy Decision-Making Among Young Black Women ‘Looked After’ By the State. Midwifery.

[38] Ochoa S.C., Sampalis J. (2014). Risk Perception and Vulnerability To STIs And HIV/AIDS Among Immigrant Latin-American Women In Canada. Cult. Health Sex..

[39] Tarzia L., Douglas H., Sheeran N. (2021). Reproductive Coercion and Abuse Against Women from Minority Ethnic Backgrounds: Views of Service Providers in Australia. Cult. Health Sex..

[40] Balaam M.C., Kingdon C., Thomson G., Finlayson K., Downe S. (2016). ‘We Make Them Feel Special’: The Experiences of Voluntary Sector Workers Supporting Asylum Seeking And Refugee Women During Pregnancy And Early Motherhood. Midwifery.

[41] Byrskog U., Olsson P., Essén B., Allvin M.K. (2015). Being a Bridge: Swedish Antenatal Care Midwives’ Encounters with Somali-Born Women and Questions of Violence; A Qualitative Study. BMC Pregnancy Childbirth.

[42] Mehta P., Gagnon A.J. (2016). Responses of International Migrant Women to Abuse Associated with Pregnancy. Violence Against Women.

[43] Rogstad K.E., Dale H. (2004). What Are the Needs of Asylum Seekers Attending an STI Clinic and Are They Significantly Different From Those Of British Patients?. Int. J. STD AIDS.

[44] Johnson-Agbakwu C.E., Manin E. (2020). Sculptors Of African Women’s Bodies: Forces Reshaping The Embodiment Of Female Genital Cutting In The West. Arch. Sex. Behav..

[45] McLeish J., Redshaw M. (2021). ‘She Come Like a Sister to Me’: A Qualitative Study Of Volunteer Social Support For Disadvantaged Women In The Transition To Motherhood In England. Philos. Trans. R. Soc. B.

[46] Ashby B.D., Ehmer A.C., Scott S.M. (2019). Trauma-Informed Care in A Patient-Centered Medical Home For Adolescent Mothers And Their Children. Psychol. Serv..

[47] Decker M.R., Flessa S., Pillai R.V., Dick R.N., Quam J., Cheng D., McDonald-Mosley R., Alexander K.A., Holliday C.N., Miller E. (2017). Implementing Trauma-Informed Partner Violence Assessment in Family Planning Clinics. J. Women’s Health.

[48] Decker M.R., Tomko C., Wingo E., Sawyer A., Peitzmeier S., Glass N., Sherman S.G. (2018). A Brief, Trauma-Informed Intervention Increases Safety Behavior and Reduces HIV Risk for Drug-Involved Women Who Trade Sex. BMC Public Health.

[49] Domoney J., Fulton E., Stanley N., McIntyre A., Heslin M., Byford S., Bick D., Ramchandani P., MacMillan H., Howard L.M. (2019). For Baby’s Sake: Intervention Development And Valuation Design Of A Whole-Family Perinatal Intervention To Break The Cycle Of Domestic Abuse. J. Fam. Violence.

[50] Hill A.L., Zachor H., Jones K.A., Talis J., Zelazny S., Miller E. (2019). Trauma-Informed Personalized Scripts to Address Partner Violence And Reproductive Coercion: Preliminary Findings From An Implementation Randomized Controlled Trial. J. Women’s Health.

[51] Kachingwe O.N., Anderson K., Houser C., Fleishman J.L., Novick J.G., Phillips D.R., Aparicio E.M. (2019). “She Was There Through the Whole Process:” Exploring How Homeless Youth Access and Select Birth Control. Child. Youth Serv. Rev..

[52] McNiel M., Held T., Busch-Armendariz N. (2014). Creating an Interdisciplinary Medical Home for Survivors of Human Trafficking. Obstet. Gynecol..

[53] Miller E., McCauley H.L., Decker M.R., Levenson R., Zelazny S., Jones K.A., Anderson H., Silverman J.G. (2017). Implementation of A Family Planning Clinic–Based Partner Violence and Reproductive Coercion Intervention: Provider and Patient Perspectives. Perspect. Sex. Reprod. Health.

[54] Salvador J.G., Bonham C.A., Duran D.K., Crisanti A.S. (2020). Impact of Seeking Safety Dose on Depression and PTSD Symptoms Among Pregnant And Post-Partum Women. J. Child Fam. Stud..

[55] Stevens N.R., Holmgreen L., Hobfoll S.E., Cvengros J.A. (2020). Assessing Trauma History in Pregnant Patients: A Didactic Module and Role-Play for Obstetrics And Gynecology Residents. MedEdPORTAL.

[56] Myers B., Carney T., Browne F.A., Wechsberg W.M. (2018). Development of A Trauma-Informed Substance Use and Sexual Risk Reduction Intervention For Young South African Women. Patient Prefer. Adherence.

[57] Myers B., Carney T., Browne F.A., Wechsberg W.M. (2019). A Trauma-Informed Substance Use And Sexual Risk Reduction Intervention For Young South African Women: A Mixed-Methods Feasibility Study. BMJ Open.

[58] Powers C., Comfort M., Lopez A.M., Kral A.H., Murdoch O., Lorvick J. (2017). Addressing Structural Barriers to HIV Care Among Triply Diagnosed Adults: Project Bridge Oakland. Health Soc. Work.

[59] Kalokhe A.S., Riddick C., Piper K., Schiff J., Getachew B., del Rio C., Sales J.M. (2020). Integrating Program-Tailored Universal Trauma Screening into HIV Care: An Evidence-Based Participatory Approach. AIDS Care.

[60] Kappel R., Lemke M., Tuchman L.K., Deye K. (2020). Featured Counter-Trafficking Program: The Cares Clinic, A Primary Care Medical Home for Commercially Exploited Youth. Child Abus. Negl..

[61] Piper K.N., Schiff J., Riddick C., Getachew B., Farber E.W., Kalokhe A., Sales J.M. (2021). Barriers and Facilitators to Implementation Of Trauma Support Services At A Large HIV Treatment Center In The Southern United States. AIDS Care.

[62] O’Connor A., Harris E., Hamilton D., Fisher C., Sachmann M. (2021). The Experiences of Pregnant Women Attending a Specialist Service And Using Methamphetamine. Women Birth: J. Aust. Coll. Midwives.

[63] Rowe H., Sperlich M., Cameron H., Seng J. (2014). A Quasi-Experimental Outcomes Analysis of a Psychoeducation Intervention For Pregnant Women with Abuse-Related Posttraumatic Stress. J. Obstet. Gynecol. Neonatal Nurs..

[64] Bargeman M., Abelson J., Mulvale G., Niec A., Theuer A., Moll S. (2022). Understanding the Conceptualization and Operationalization Of Trauma-Informed Care within And Across Systems: A Critical Interpretive Synthesis. Milbank Q..

[65] Goldstein E., Chokshi B., Melendez-Torres G., Rios A., Jelley M., Lewis-O’Connor A. (2024). Effectiveness of Trauma-Informed Care Implementation In Health Care Settings: Systematic Review Of Reviews And Realist Synthesis. Perm. J..

[66] Kaufman R., Brown R., Martínez Coral C., Jacob J., Onyango M., Thomasen K. (2022). Global Impacts of Dobbs V. Jackson Women’s Health Organization and Abortion Regression in The United States. Sex. Reprod. Health Matters.

[67] Nguyen D., Bajaj S.S., Ahmed D., Stanford F.C. (2022). Protecting Marginalized Women’s Mental Health in The Post-Dobbs Era. Proc. Natl. Acad. Sci. USA.

[68] Raja S., Rabinowitz E.P., Gray M.J. (2021). Universal Screening and Trauma Informed Care: Current Concerns And Future Directions. Fam. Syst. Health.

[69] Coughlin M.E., Kenner C. (2020). The Value Proposition of Trauma-Informed Care. Transformative Nursing in the NICU.

[70] Williamson L.F., Kautz D.D. (2016). Trauma-Informed Care Is the Best Clinical Practice in Rehabilitation Nursing. Rehabil. Nurs. J..

[71] Agency for Healthcare Research and Quality (AHRQ) (2014). Community-Driven Clinic for Refugee Women Enhances Access to Comprehensive, Culturally Sensitive Care across the Reproductive Life Span. U.S. Department of Health and Human Services. https://web.archive.org/web/20170130235937/https://innovations.ahrq.gov/profiles/community-driven-clinic-refugee-women-enhances-access-comprehensive-culturally-sensitive.

[72] Banke-Thomas A., Gieszl S., Johnson-Agbakwu C. (2017). Experiences of Refugee Women in Accessing And Utilizing A Refugee-Focused Prenatal Clinic In The United States: A Mixed Methods Study. Glob. Women’s Health.

[73] Schuster R.C., Wachter K., McRae K., McDaniel A., Davis O.I., Nizigiyimana J., Johnson-Agbakwu C.E. (2024). “If You Don’t Have the Heart To Help, You Cannot Do This Job”: The Multidimensional Wellbeing Of Community Health Workers Serving Refugees During The COVID-19 Pandemic. Qual. Health Res..

[74] Johnson-Agbakwu C.E., Eakin C.M., Bailey C.V., Sood S., Ali N., Doehrman P., Bhattarai B., Chambliss L., Coonrod D.V. (2021). Severe Acute Respiratory Syndrome Coronavirus 2: A Canary in The Coal Mine for Public Safety Net Hospitals. AJOG Glob. Rep..

[75] Johnson-Agbakwu C.E., Fox K.A., Banke-Thomas A., Michlig G.J. (2023). Influence of Female Genital Mutilation/Cutting on Health Morbidity, Health Service Utilization and Satisfaction with Care among Somali Women And Teenage Girls in the United States. J. Racial Ethn. Health Disparities.

[76] Michlig G., Warren N., Berhe M., Johnson-Agbakwu C. (2021). Female Genital Mutilation/Cutting Among Somali Women in The U.S. State of Arizona: Evidence Of Treatment Access, Health Service Use And Care Experiences. Int. J. Environ. Res. Public Health.

[77] Michlig G.J., Johnson-Agbakwu C., Surkan P.J. (2022). “Whatever You Hide, Also Hides You”: A Discourse Analysis on Mental Health and Service Use In An American Community Of Somalis. Soc. Sci. Med..

[78] Jolof L., Rocca P., Carlsson T. (2024). Trauma-Informed Care for Women Who Are Forced Migrants: A Qualitative Study Among Service Providers. Scand. J. Public Health.

[79] Badanta B., González-Cano-Caballero M., Fernández-García E., Lucchetti G., de Diego-Cordero R. (2023). The Consequences of The COVID-19 Pandemic On The Refugee Population: A Rapid Review. Perspect. Public Health.

[80] Samari G., Wurtz H.M., Desai S., Coleman-Minahan K. (2024). Perspectives from The Pandemic Epicenter: Sexual And Reproductive Health Of Immigrant Women In New York City. Perspect. Sex. Reprod. Health.

